# Prediction of Novel Ultrahard Phases in the B–C–N System from First Principles: Progress and Problems

**DOI:** 10.3390/ma16020886

**Published:** 2023-01-16

**Authors:** Vladimir L. Solozhenko, Samir F. Matar

**Affiliations:** 1 LSPM–CNRS, Université Sorbonne Paris Nord, 93430 Villetaneuse, France; 2 CMMS (Computational Materials & Molecular Science), Lebanese German University (LGU), Jounieh P.O. Box 206, Lebanon

**Keywords:** B–C–N system, DFT, crystal structure, elastic moduli, hardness

## Abstract

The modern synthesis of superhard and, especially, ultrahard phases is a fascinating area of research that could lead to the design of new, industrially important materials. Computational methods built within the well-established quantum mechanics framework of density functional theory (DFT) play an important role in the search for these advanced materials and the prediction of their properties. The close relationship between the physical properties of carbon and boron nitride has led to particular interest in the B–C–N ternary system, characterized by the small radii of the elements, resulting in short interatomic distances and reduced volumes—the parameters being ‘recipes’ for very high hardness in three-dimensional structures. The purpose of this review is to provide a brief outline of recent developments and problems in predicting novel ultrahard carbon allotropes as well as binary and ternary compounds of the B–C–N system with particular emphasis on the analysis of the models used to evaluate the hardness of the theoretically predicted structures.

## 1. Introduction

Historically, research in the field of ultrahard materials (usually defined as having Vickers hardness *H*_V_ ≥ 80 GPa) was initiated a century ago, triggered by the increasing industrial application of diamond, the hardest (*H*_V_ up to 120 GPa) known material. However, the use of diamond in mining and tooling raised problems due to the high cost of natural diamond, on the one hand, and its relatively low stability at even moderate operating temperatures, on the other hand. The first problem was solved by the development of the high-pressure synthesis of diamond [1], but its high reactivity with oxygen and ferrous metals remained a problem that required further efforts to find reliable substitutes for diamond.

An interim solution was the synthesis of cubic boron nitride (cBN) [2], which is half as hard as diamond, but has much higher thermal and chemical stability. It should be noted that in terms of electronic structure, BN is equivalent to 2C (*vide infra*). The close relationship between the physical properties of carbon allotropes and BN polymorphs has facilitated the search for ultrahard phases in the B–C–N ternary system constituted by light elements with small radii, resulting in short interatomic distances and reduced volumes—all the parameters being ‘recipes’ for high strength and hardness.

Until very recently, diamond was the only known material that was ultrahard. However, in 2001, cubic BC_2_N, a ternary compound that is halfway between diamond and BN in composition, was synthesized [3]. Vickers hardness of 76(4) GPa [4] makes it the second member of the ultrahard phases family. In 2009, the third ultrahard phase, diamond-like BC_5_, was discovered [5], which possesses Vickers hardness of 71(8) GPa, an unusually high for superhard materials fracture toughness (~10 MPa·m^½^), and very high (up to 1900 K) thermal stability. Both novel ultrahard phases have been synthesized at very high pressures and temperatures with in situ control using synchrotron X-ray diffraction, which indicates that the experimental search for such phases is a challenging task [6]. 

Since experimental material discovery is suffering from the high labor costs and limitations inherent to trial-and-error methods, theoretical approaches for predicting the mechanical properties of solids have been developed, from empirical models of hardness [7,8,9,10] to the ab initio calculation of elastic constants [11,12] and computational discovery of superhard materials [13,14,15]. Thus, modern algorithms and powerful computers can be used to search for materials with exceptional mechanical properties. 

Studies within the B–C–N ternary system are a growing area of exploration, complementing the competence of experimenters using modern low- and high-pressure techniques, well adapted to light elements with high-purity sources, and theorists employing structure research tools such as CALYPSO [16] and USPEX [17,18] to access accurate characterizations of electronic structures and energy-related quantities such as mechanical and dynamic signatures from quantum mechanics software built in the framework of density functional theory (DFT) (cf. Appendix A). Calculation results serve as both interpretive and predictive tools for new materials with desired properties. 

**Figure 1:**
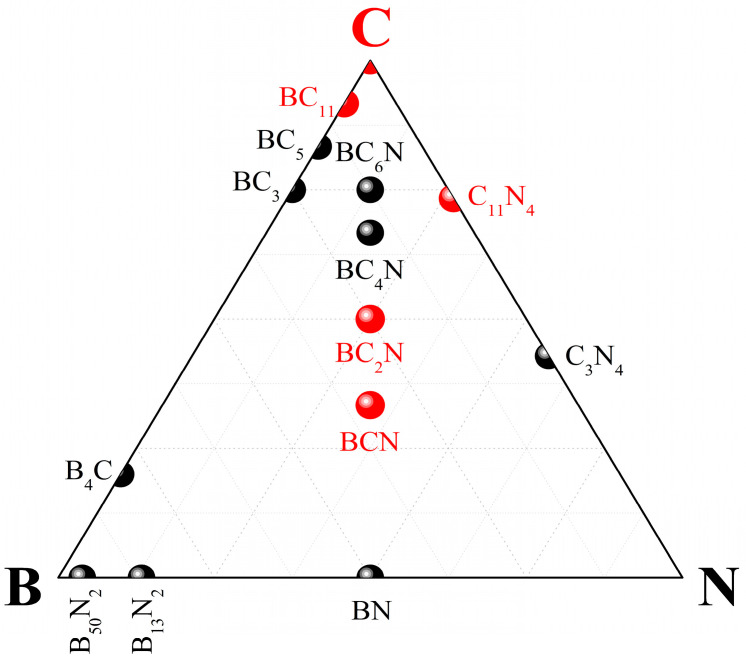
Superhard phases of the B–C–N ternary system. Ultrahard phases discussed in the present paper are shown in red.

Since the present paper is not intended to be a comprehensive and exhaustive review, we aim to focus on the presentation of the recent developments and problems in this emerging research field, and illustrate the subject using examples from our own studies (both published and unpublished) (Figure 1). To highlight the structural and physical properties in the main body of review, we present the computational framework in Appendix A.

## 2. Elements

Carbon and boron (in particular, their dense allotropes, i.e., diamond and γ-B_12_ [19]) are the hardest elements. We shall not discuss boron here (the problem of searching for its superhard forms was already addressed in a recent review [20]), but will rather concentrate on carbon. The main research efforts with respect to carbon are focused on the search for new, dense allotropes with mechanical properties close to those of diamond. Diamond is known to exist in two forms: the cubic one that belongs to space group *Fm*−3*m* (No. 227), and a rare hexagonal form called ‘lonsdaleite’ (space group *P*6_3_/*mmc*, No. 194). Recently, however, the existence of lonsdaleite as a discrete phase has been questioned, and it has been interpreted as a cubic diamond dominated by extensive twins and stacking faults [21]. 

It should be noted that information on 3-periodic carbon allotropes extracted from the scientific literature is gathered and indexed in the “SACADA” database [22]. Currently, it counts 524 unique carbon allotropes. From the structural point of view, and using chemistry terms, diamond is characterized by tetrahedral (sp^3^) carbon, as in the methane gas molecule CH_4_, and its crystal structure demonstrates three-dimensional stacking of corner-sharing *C4* tetrahedra. In contrast, graphite is characterized by a layered structure where carbon is sp^2^ hybridized, as in the ethene gas molecule C_2_H_4_. While diamond is a large-band-gap insulator with E_gap_ ≈ 5 eV, graphite is a semiconductor with a very small gap. Mixed sp^2^-sp^3^ hybridizations carbon was recently reported for a new, stable metallic allotrope, *hex*-C_18_ (called 18*H* carbon), which belongs to space group *P*6/*mmm* (No. 191) [23]. 

**Figure 2:**
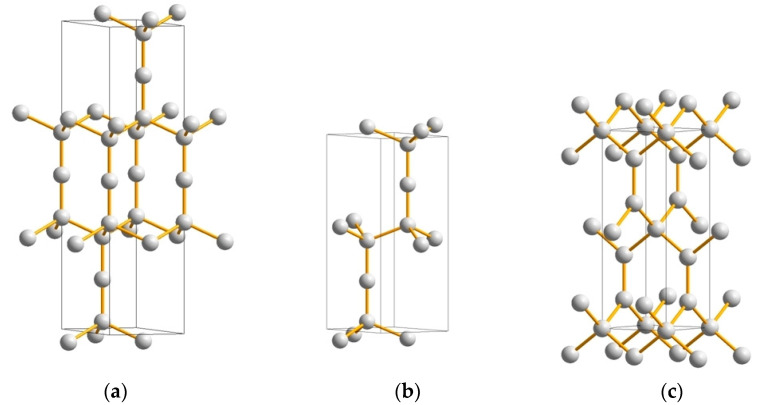
Novel ultrahard carbon allotropes: ‘tricarbons’—rhombohedral C_3_ (**a**) and hexagonal C_6_ (**b**) (both structures are in hexagonal setting); and tetragonal C_6_ ‘neoglitter’ (**c**).

Carbon in a linear triatomic C–C–C arrangement is found in carbon suboxide C_3_O_2_, where carbon is sp^2^ hybridized. In the solid state, the linear configuration is kept for carbon suboxide, which is a molecular solid where weak (van-der-Waals-like) interactions prevail between separate molecules. The rarely occurring tricarbon C_3_ molecule was observed in interstellar space, mainly in the tails of comets (e.g., Hale-Bopp, C/1995O1), and experimentally identified by spectroscopic measurements [24]. Recently [25], we considered linear C–C–C in two novel structures: (i) *rh*-C_3_ (or *hex*-C_9_) (space group *R*−3*m*, No. 166) based on rhombohedral sodium azide characterized by the presence of a linear N_3_ fragment (Figure 2a), and (ii) *hex*-C_6_ (space group *P*6_3_/*mmc*, No. 194) derived from lonsdaleite through the insertion of one extra carbon atom along the *c*-axis at 2d (2/3,1/3,¼) Wyckoff position (Figure 2b). From geometry optimization to the energy ground state within DFT (cf. Appendix A), energies and the energy-derived quantities were found within the range of diamond and lonsdaleite.

The elastic properties of ‘tricarbon’ allotropes *rh*-C_3_ and *hex*-C_6_ were determined by performing finite distortions of their lattices. The elastic constants C_ij_ were derived from the strain–stress relationship. Indexes i and j represent directions: when i = j, the elastic constants correspond to the application of unidirectional stress (as in the case of C_33_ elastic constant discussed below), and when i ≠ j, the elastic constants are relevant to applying shear stress. For both allotropes, all calculated C_ij_ values are positive, and their combinations obey the rules pertaining to the mechanical stability of the phases. The bulk (*B*_V_) and shear (*G*_V_) moduli were calculated from the elastic constants following the Voigt method [26], based on a uniform strain.

The Vickers hardness of the new carbon allotropes was predicted using four modern theoretical models [8,19,27,28]. The thermodynamic model [27] is based on the thermodynamic properties and crystal structure, the empirical Chen–Niu [8] and Mazhnik–Oganov [10] models use the elastic properties, and the Lyakhov–Oganov approach [28] considers the topology of the crystal structure, strength of covalent bonding, degree of ionicity and directionality. The fracture toughness (K_Ic_) was evaluated within the Mazhnik–Oganov model [10]. Table 1 and Table 2 present the hardness values calculated using all four models, and other mechanical properties such as the bulk (*B*), shear (*G*) and Young’s (*E*) moduli, the Poisson’s ratio (ν) and the fracture toughness (K_Ic_).

As has been reported earlier [36], in the case of ultrahard compounds of light elements, the thermodynamic model shows surprising agreement with available experimental data. Moreover, its use is preferable in the case of hybrid dense carbon allotropes, for which the Lyakhov–Oganov model gives underestimated hardness values, whereas the empirical models are not reliable. For this reason, both new ‘tricarbon’ allotropes should be considered as ultrahard phases.

Although the hardness and elastic moduli of *rh*-C_3_ and *hex*-C_6_ are somewhat lower than those of diamond, strong anisotropy was found for both ‘tricarbon’ allotropes, with exceptionally large C_33_ values along the hexagonal *c*-axis, i.e., C_33_ = 1636 GPa for *rh*-C_3_ (Figure 2a) and C_33_ = 1610 GPa for *hex*-C_6_ (Figure 2b), exceeding the corresponding value for lonsdaleite, C_33_ = 1380 GPa. The Vickers hardness predicted using four theoretical models (Table 1 and Table 2) points to slightly lower *H*_V_ values for these new carbon allotropes compared to diamond (both cubic and hexagonal), but much higher than the hardness of the vast majority of recently predicted carbon allotropes, such as C_14_, C_16_, C_24_, C_36_, etc. [42,43,44,45]. Thus, both *rh*-C_3_ and *hex*-C_6_ have exceptional mechanical properties and can be considered as prospective ultrahard phases [46].

The dynamic stability of the ‘tricarbon’ allotropes was confirmed by phonon calculations. All frequencies are positive, with the particular feature of a gap in the highest-frequency optical phonon domain, not observed in lonsdaleite, and caused by the rigidly aligned C_3_ unit. A remarkable consequence of the presence of two carbon hybridizations (sp^2^ and sp^3^) is the occurrence of a metallic character in the electronic band structure of both ‘tricarbon’ allotropes, similar to that previously observed for hexagonal C_18_ [23] and monoclinic C_12_ [47] characterized by mixed sp^2^-sp^3^ and sp^1^-sp^2^ hybridizations, respectively.

Another novel ultrahard carbon allotrope, body-centered tetragonal C_6_ (space group *I*−4*m*2, No. 119) presenting mixed sp^2^/sp^3^ hybridizations, has been proposed very recently via a crystal chemistry approach and studied for the ground state structure and stability using DFT calculations [29]. Since *C4* tetrahedra are in-plane stacked with corner-sharing and connected out-of-plane with C–C trigonal carbon (Figure 2c), a close relationship with so-called ‘glitter’, a hypothetical dense carbon network invented in 1994 [48], is apparent, and thus the new allotrope was named ‘neoglitter’. Besides the mechanical stability (positive values of elastic constants and their combinations), ‘neoglitter’ is also dynamically stable, as it follows from its phonon band structure. The novel allotrope reveals exceptional mechanical properties, i.e., very high hardness and elastic moduli (see Table 1 and Table 2), being conductive due to the metallic-like electronic structure, which is mainly caused by the itinerant role of trigonal carbon π-electrons.

Since the Vickers hardness calculated in the framework of the thermodynamic model exceeds 80 Gpa for the three novel carbon allotropes described above, they all should be attributed to the family of ultrahard phases.

## 3. Binary Compounds

The hardness of dense compounds of the binary B–N system—cubic BN [2], rhombohedral B_13_N_2_ [49,50] and tetragonal B_50_N_2_ [51]—does not exceed *H*_V_ = 62 Gpa for single-crystal cBN [4], i.e., they all belong to the group of superhard phases. Special mention should be given to nanocrystalline cBN [52], with Vickers hardness up to 85 Gpa [53], mainly due to the Hall–Petch effect, i.e., nanosize effect, which restricts dislocation propagation through the material.

We will focus in more detail on two other binary systems, i.e., C–N and B–C, in which compounds with very high hardness have been predicted.

### 3.1. Carbon Nitrides

The main interest in studying the C–N system is a result of numerous (but unsuccessful) attempts to synthesize hypothetical ultrahard C_3_N_4_. In the 1990s, Liu et al. [30,54] and Teter and Hemley [31] predicted a number of dense low-compressibility carbon nitrides of C_3_N_4_ stoichiometry that were claimed to exhibit bulk moduli and hardness higher than those of diamond because of the short length and high covalence of the C–N bonds. However, our analysis in the framework of the thermodynamic model of hardness reveals that the Vickers hardness of the densest hypothetical cubic (*P*−43*m* [30] and *I*−43*d* [31]) and pseudocubic (*P*−42*m* [31]) polymorphs of C_3_N_4_ does not exceed 73 GPa.

Besides the carbon nitrides of C_3_N_4_ stoichiometry that are isoelectronic with diamond [55], carbon subnitrides of C_11_N_4_ stoichiometry were also studied [56] as they allow the modeling of CN_x_ films with less than 30 at% N, which usually form when vapor phase deposition techniques are used [57]. In this context, structural models of C_11_N_4_ phases accounting for the low nitrogen content were derived from diamond being isoelectronic with it, through creating defects. Indeed, diamond expressed as 2C in primitive cells has eight valence electrons, and C_11_N_4_ has 11 × 4 + 4 × 5 = 64 = 8 × 8 electrons, i.e., an integer multiple of 8.

**Figure 3:**
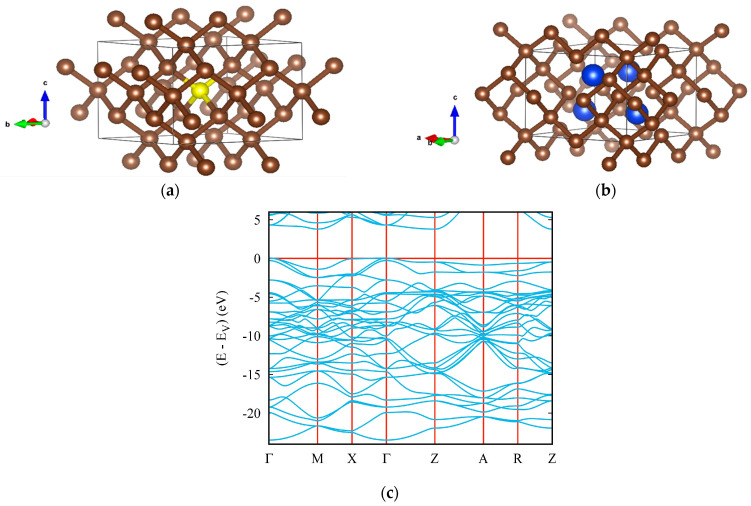
Novel ultrahard carbon subnitride: crystal structures of template diamond-like C_16_ (**a**) and tetragonal C_11_N_4_ (**b**); electronic band structure of C_11_N_4_ (**c**).

In the present paper, a novel (ultra)hard tetragonal C_11_N_4_ (space group *P*−4*m*2, No. 115) was derived from C_16_, a 2 × 2 × 1 cell of body-centered tetragonal C_4_ diamond-like structure [58] (Figure 3a). In this template, a defect was created at the center of the tetragonal cell by removal of the yellow carbon atom (see Figure 3a) and replacing the four surrounding carbon atoms with nitrogen (blue spheres in Figure 3b). The resulting fully relaxed carbon subnitride C_11_N_4_ was analyzed for the cohesive energy E_coh_ obtained from subtracting the atomic energies of 11 C and 4 N atoms from the calculated total energy. C_11_N_4_ was found to be cohesive, with E_coh_ = −1.93 eV/atom, which is lower than the corresponding value for pristine C_16_ (E_coh_ = −2.49 eV/atom, the value identifying diamond). This is quite expected, since C_11_N_4_ results from the defect diamond-like structure of C_16_. Such observations are also valid for other binary compounds resulting from the perturbation of the diamond lattice.

The lattice parameters of C_11_N_4_ are *a* = *b* = 4.952 Å and *c* = 3.520 Å, with a nitrogen atom occupying the 4k site (0.50000, 0.24200, 0.72825) and four inequivalent carbon atoms located at the 1a (0.00000, 0.00000, 0.00000), 2g (0.50000, 0.00000, 0.97312), 4i (0.25368, 0.25368, 0.50000) and 4j (0.24974, 0.00000, 0.23750) sites. Besides being cohesive, C_11_N_4_ was found to be mechanically stable, with the whole set of elastic constants being positive, as well as being dynamically stable, with positive acoustic and optic phonon frequencies.

Calculated values of the hardness and elastic moduli of *tet*-C_11_N_4_ are listed in Table 1 and Table 2. Although the bulk and shear moduli of the novel carbon subnitride are lower than the corresponding values for high-density C_3_N_4_, its Vickers hardness *H*_V_ = 76 GPa is higher than that of all hypothetical high-density polymorphs of C_3_N_4_ [30,31,54].

The electronic band structure of the novel tetragonal C_11_N_4_ is shown in Figure 3c, characterizing an insulator with a gap value slightly below 5 eV, from Γ (valence band) to Z (conduction band), similar to that of diamond, as a result of both structures being isoelectronic, as mentioned above.

### 3.2. Boron Carbides

Rhombohedral boron carbide, B_4_C (B_12_C_3_), is the most important, well-studied and widely used compound of the B–C binary system; however, its Vickers hardness does not exceed 37 Gpa [59]. The synthesis of diamond-like BC_5_ with hardness above 70 GPa [5] has stimulated interest in the study of carbon-rich compounds of this system. Thus, four polymorphs of BC_5_ were predicted from first-principles structural optimizations [32], for two of which (tetragonal *I*−4*m*2 and triclinic *P*−1) Vickers hardness of approximately 80 Gpa was claimed. However, our assessments (the results for the densest tetragonal BC_5_ are presented in Table 1 and Table 2) showed that the hardness of all predicted phases was overestimated by ~15% as a result of the use of unreliable empirical correlations between the shear modulus and hardness.

A similar situation is observed in the case of five predicted BC_7_ polymorphs [33]: the claimed hardness values (e.g., 81 GPa for orthorhombic *Pmm*2 and 78 GPa for cubic *P*−43*m* phases) are also overestimated by 10–15% as a result of using the empirical microscopic hardness model (see our results for cubic BC_7_ in Table 1 and Table 2), and thus these phases, as well as BC_5_ polymorphs, cannot be considered ultrahard. However, one may expect that a further decrease in boron content will be accompanied by a hardness increase in the formed B-C binary compound(s).

Very recently, the latter has been confirmed by the prediction of trigonal BC_11_ (space group *P*3*m*1, No. 156) [34], produced by the substitution of carbon with boron in the diamond-like *hex*-C_12_ template [60], which led to the lowering of the crystal symmetry down to trigonal.

In the context of the energy criterion, it was interesting to position the novel carbon-rich BC_11_ among other B–C binary compounds. Comparison of *trig*-BC_11_’s cohesive energy with those reported for *trig*-BC_5_ [32] and trig-BC_7_ [33] shows a clear trend of decreasing E_coh_/atom with increasing boron content: −2.49 eV (diamond) < −2.33 eV (BC_11_: 8.3 at% B) < −2.24 eV (BC_7_: 12.5 at% B) < −2.16 eV (BC_5_: 16.7 at% B). Besides being more cohesive than the two other binary compounds, *trig*-BC_11_ was also found to be mechanically (elastic constants) and dynamically (phonon band structures) stable.

**Figure 4:**
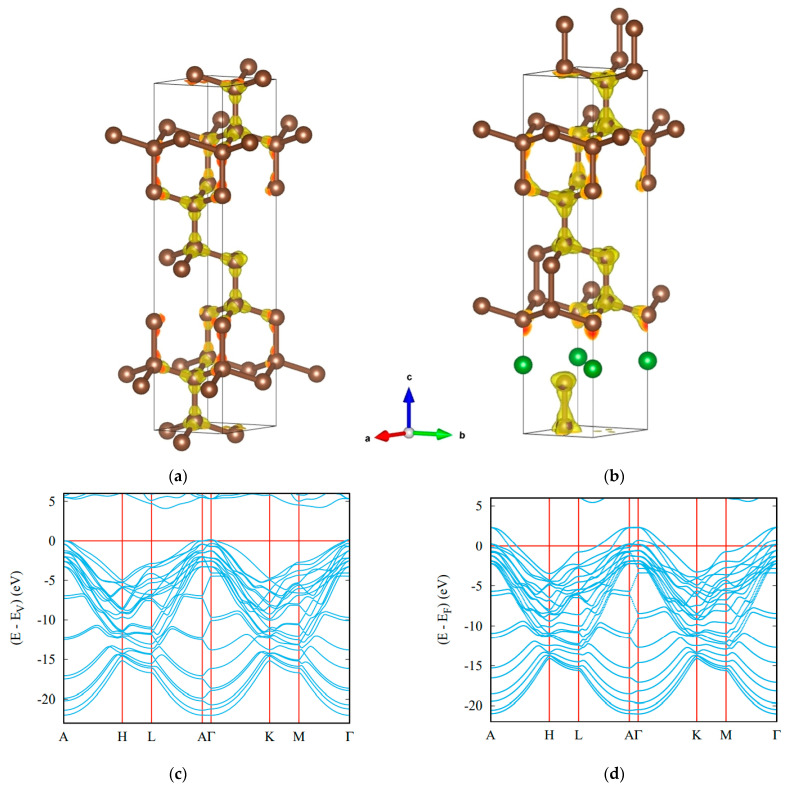
Ultrahard carbon-rich boron carbide: crystal structures of template hexagonal C_12_ (**a**) and trigonal BC_11_ (**b**) with charge density distributions, and electronic band structures of insulating *hex*-C_12_ (**c**) and metallic BC_11_ (**d**). Boron atoms are shown by green spheres.

The crystal structures of template *hex*-C_12_ and *trig*-BC_11_ are shown in Figure 4. In *hex*-C_12_, the carbon network is perfectly covalent in all dimensions. Changes are observed for *trig*-BC_11_, featuring a large covalent part where the carbon networks remain as in *hex*-C_12_, but not in the surrounding area of boron atoms, whose charge density is transferred to carbon due to the larger electronegativity of carbon. Regarding its electronic band structure, bands belonging to boron states were found crossing the Fermi level E_F_, signaling a metallic character arising from one electron-less B (2s^2^,2p^1^) versus C (2s^2^,2p^2^) in the wide gap insulating diamond.

The hardness of *trig*-BC_11_ calculated using four models, as well as other mechanical properties, is given in Table 1 and Table 2. Although the introduction of boron atoms into the diamond crystal lattice predictably lowers the hardness, it remains high enough (>80 GPa) to consider *trig*-BC_11_ as an ultrahard phase, in contrast to other reported binary compounds of the B–C system [32,33,61].

## 4. Ternary Compounds

Interest in the search for possible ‘hybrid’ structures of carbon and boron nitride and prediction of their properties (mechanical, in particular) has especially grown after the synthesis of ultrahard cubic BC_2_N [3]. Over the past 20 years, several dozen papers have been published on the subject, but here we will focus only on those that claim the ‘ultrahardness’ (*H*_V_ ≥ 75 GPa) of the predicted phases. In addition to different BC_2_N structures [35,36,62,63], ultrahard phases of BCN [64], BC_4_N [65,66,67], BC_6_N [68,69] and BC_10_N [70] compositions have been reported.

**Figure 5:**
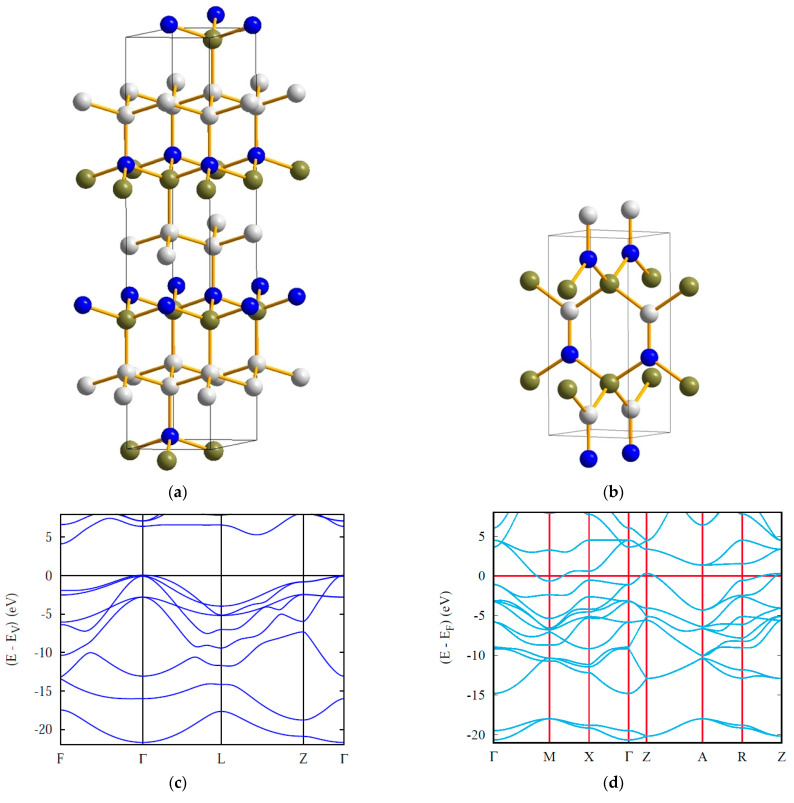
Crystal structures (**a**,**b**) and electronic band structures (**c**,**d**) of rhombohedral BC_2_N and tetragonal BCN. Boron, carbon and nitrogen atoms are shown by olive, white and blue spheres, respectively.

Our assessments for orthorhombic (*P*222_1_) and trigonal (*P*3*m*1) BC_2_N [35], as well as for novel rhombohedral (*R*3*m*) BC_2_N [36] (Figure 5a), show that they all have Vickers hardness of the order of 75 GPa (see Table 1 and Table 2; the *H*_V_ values calculated using the thermodynamic model are the most reliable), i.e., almost the same as experimental value 76(4) GPa for cubic BC_2_N [3,4]. As for the 79.7 GPa hardness claimed for the calculated low-energy zinc-blended BC_2_N [63], it seems to be overestimated due to the use of the empirical hardness model suggested by Gao et al. [71] (see Table 3).

A similar situation is observed for trigonal (*P*3*m*1) [65] and orthorhombic (*Imm*2) [66] BC_4_N, and tetragonal (*P*−42*m*) [68]*,* rhombohedral (*R*3*m*) [68] and monoclinic (*Pm* and *Cm*) [69] BC_6_N. In all these cases, the use of Gao’s model results in a 4–11% hardness overestimation compared to the values that we obtained in the framework of the thermodynamic model of hardness (see Table 3).

The use of another empirical so-called ‘microscopic’ model of hardness suggested by Tian et al. [9] for trigonal BC_4_N [67] and BC_10_N [70] leads to even higher (15–20%) overestimations of hardness compared to the thermodynamic model (see Table 3).

In general, according to our estimates, the hardness of the dense ternary phases of the aforementioned compositions varies in the 72–76 GPa range, i.e., they all can be considered as (ultra)hard. Regarding possible equiatomic (B:C:N = 1:1:1) phases, their hardness should be even lower (~70 GPa); see, e.g., *H*_V_ values for trigonal (*P*−3*m*1) and tetragonal (*I*4_1_*md*) BCN [64] (Table 3).

The electronic band structure of rhombohedral BC_2_N [36] (Figure 5c) is characteristic of an insulator, similar to diamond. In fact, they both have a valence electron count multiple of 8 (C_2_), i.e., 16 (2 × 8) for BC_2_N and 32 (4 × 8) for diamond (C_8_).

Below, we discuss in more detail the tetragonal (*P*4_2_*mc*) equiatomic boron carbonitride (Figure 5b) recently proposed by us using crystal chemistry rationale and DFT calculations [37].

Compounds containing CN^−^ anions (such as ionic sodium cyanide Na^1+^CN^1−^) are called ‘cyanides’. Since boron is a metalloid (i.e., halfway between a metal and non-metal), its combination with nitrogen leads to the equiatomic boron nitride BN that could be expressed as ‘B^3+^N^3−^’ considering its ionic-like nature. However, BN is rather a polar covalent compound, as can be inferred from the Pauling electronegativity difference (Δχ). Considering the average electronegativity of CN, <χ(CN)> = (2.55 + 3.44)/2 ~ |3.0|, NaCN has Δχ = 0.9 − 3.0 = |2.1|, whereas BN is characterized by Δχ = 2.04 − 3.44 = |1.4|. For the presently proposed BCN, Δχ = 2.04 − 3.0 = |0.96|, which indicates a decrease in ionic character in the NaCN → BN → BCN row.

**Figure 6:**
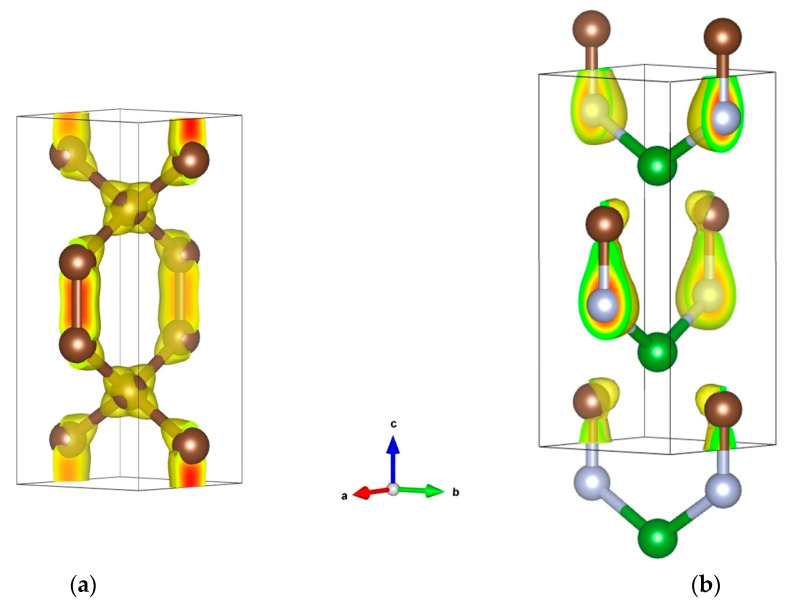
Crystal structures of template ‘glitter’ C_6_ (**a**) and tetragonal BCN (**b**) with charge density distributions. Boron, carbon and nitrogen atoms are shown by green, brown and grey spheres, respectively.

In the framework of the crystal chemistry approach, we considered three template structures for BCN: octahedral (CoCN), square-planar (NaCN) and linear (CuCN). The square-planar *BC2N2* coordination was found to be the most stable among all three templates in terms of cohesive energy, but despite the relative stability, it remains a non-compact 2D-like structure. Therefore, as a 3D template allowing tetrahedral coordination for B with C and N, and connected *BC2N2* tetrahedra, we used tetragonal hexacarbon C_6_, so-called ‘glitter’ [48], which possesses two types of carbon, tetrahedral C1 and trigonal C2, the latter forming C2–C2 pairs that separate the *C1C2_4_* tetrahedra. The structure shown in Figure 6a featuring the charge density projections reveals the two types of carbons in the ‘glitter’ structure, and the corresponding *C1C2_4_* tetrahedra (C1: sp^3^-like carbon). With appropriate substitutions of carbon for boron and nitrogen leading to BCN, the ground state energy configuration of the derived 3D structure was found to be more cohesive than the 2D-like candidate mentioned above.

The resulting BCN (B_2_C_2_N_2_) structure sketched in Figure 6b shows *BC_2_N_2_* tetrahedra replacing the *C1C2_4_* tetrahedra in ‘glitter’ C_6_, and large differences in charge density distribution as compared to C_6_, with a charge density concentration skewed toward C–N bonds, and larger intensity on N versus C. Such B → C → N charge transfers are expected from the Pauling electronegativities: χ(B) = 2.04 < χ(C) = 2.55 < χ(N) = 3.44. In the case of BN, three electrons depart from B to N, leading to B^3+^N^3−^. In BCN, we equally observe B^3+^, but the negative ‘3-’ charge is now distributed between C and N, with a larger value on N due to its larger electronegativity versus C, and one obtains B^3+^C^0.316−^N^2.684−^. As follows from our results, cohesive tetragonal BCN is mechanically (elastic constant) and dynamically (phonon) stable. An interesting feature of this phase is that, at a relatively low density (2.783 g/cm^3^), it is characterized by very high hardness, *H*_V_ = 65 GPa (i.e., harder than single-crystal cubic boron nitride, with density of 3.486 g/cm^3^ [72]), highly likely due to the presence of both tetrahedral (sp^3^) and trigonal (sp^2^) carbons in its crystal structure. Such mixed hybridizations in tetragonal BCN lead to its weakly metallic behavior, as illustrated by the electronic band structure in Figure 5d, exhibiting a few bands crossing the Fermi level E_F_.

## 5. Conclusions

The modern high-pressure synthesis of superhard and, especially, ultrahard phases is a fascinating area of research that could lead to the production of industrially important new materials. However, this field is still in its infancy, and a large number of new super- and ultrahard phases still remain to be discovered. Theoretical predictions play an important role in the present search for advanced materials with desired properties (mechanical, in particular). In this review, we have illustrated, with selected examples, the wealth of (ultra)hard allotropes and phases in the B–C–N ternary system, the theoretical (crystal chemistry considerations combined with quantum mechanics calculations) study of which is a very active area of research.

At the same time, precise calculations of the mechanical properties of superhard materials (hardness, in particular) often lie beyond the capabilities of the most advanced and modern techniques. Thus, we should state that neither widely used empirical models [7,8,9,10,71] nor machine learning [14,70,73] allow us to reliably estimate the hardness of newly predicted superhard and, especially, ultrahard phases. The only model that seems to work in these cases is the thermodynamic model [27]. Moreover, not all theoretically predicted structures exist or can be synthesized. A vivid illustration is the case of hypothetical cubic C_3_N_4_ with a bulk modulus claimed to be higher than that of diamond [31]. Despite enormous efforts (new attempts are still being undertaken), this phase has not been synthesized so far, and its expected ultrahardness has never been demonstrated.

Finally, it should be noted that the search for new ultrahard phases is indeed at the frontier of fundamental science and promises great prospects for the creation of new materials that are needed for existing and prospective applications. However, the recent advances in this field clearly show that phases with hardness exceeding that of diamond are highly unlikely, or even impossible [40].

## Figures and Tables

**Table 1:** (Ultra)hard phases of the B–C–N system: lattice parameters, density (ρ), Vickers hardness (*H*_V_) and bulk moduli (*B*_0_) calculated in the framework of thermodynamic model of hardness [27].

	Space Group	*a = b* (Å)	*c* (Å)	ρ (g/cm^3^)	*H*_V_ (GPa)	*B*_0_ (GPa)
Diamond	*Fd*−3*m*	3.56661 ^‡^		3.517	98 ^*pw*^	445 ^§^
Lonsdaleite	*P*6_3_*/mmc*	2.5221 ^†^	4.1186 ^†^	3.516	97 ^*pw*^	443 ^*pw*^
*rh*-C_3_^#166^ [25]	*R*−3*m*	2.4900	10.4100	3.211	89	406
*hex*-C_6_^#194^ [25]	*P*6_3_/*mmc*	2.4950	6.9610	3.189	87	404
*tet*-C_6_^#119^ [29]	*I*−4*m*2	2.4666	6.4320	3.058	85	385
*c*-C_3_N_4_^#215^ [30]	*P*−43*m*	3.4300		3.788	71 ^*pw*^	421 ^*pw*^
*c*-C_3_N_4_^#220^ [31]	*I*−43*d*	5.3973		3.889	73 ^*pw*^	430 ^*pw*^
*tet*-C_11_N_4_^#115^ [*pw*]	*P*−4*m*2	4.9526	3.5202	3.618	76	408
*tet*-BC_5_^#119^ [32]	*I*−4*m*2	2.5250	11.3230	3.260	70 ^*pw*^	350 ^*pw*^
*c*-BC_7_^#215^ [33]	*P*−43*m*	3.6205		3.320	72 ^*pw*^	358 ^*pw*^
*trig*-BC_11_^#156^ [34]	*P*3*m*1	2.5381	12.5955	3.378	83 ^*pw*^	395 ^*pw*^
*o*-BC_2_N ^#17^ [35]	*P*222_1_	3.5536/3.5986	3.5528	3.570	73 ^*pw*^	381 ^*pw*^
*trig*-BC_2_N ^#156^ [35]	*P*3*m*1	2.4955	4.1923	3.587	74 ^*pw*^	384 ^*pw*^
*rh*-BC_2_N ^#160^ [36]	*R*3*m*	2.5382	12.6054	3.460	75	426 ^*pw*^
*tet*-BCN ^#105^ [37]	*P*4_2_*mc*	2.7047	6.0073	2.783	65	282

^†^ Ref. [38]; ^‡^ Ref. [39]; ^§^ Ref. [40]; *^pw^* present work.

**Table 2:** Mechanical properties of (ultra)hard phases of the B–C–N system: Vickers hardness (*H*_V_), bulk modulus (*B*), shear modulus (*G*), Young’s modulus (*E*), Poisson’s ratio (ν) and fracture toughness (*K*_Ic_).

	*H*_V_				*B*		*G*_V_	*E* **	*ν* **	*K*_Ic_^‡^
	T *	LO ^†^	MO ^‡^	CN ^§^	*B*_0_ *	*B*_V_				
	GPa									MPa·m^½^
Diamond [*pw*]	98	90	100	93	445 ^††^		530 ^††^	1138	0.074	6.4
Lonsdaleite [*pw*]	97	90	99	94	443	432	521	1115	0.070	6.2
*rh*-C_3_^#166^ [25]	89	83	73 ^*pw*^	65 ^*pw*^	406	394	402	900	0.119	5.1
*hex*-C_6_^#194^ [25]	87	82	73 ^*pw*^	65 ^*pw*^	404	392	400	895	0.119	5.1
*tet*-C_6_^#119^ [29]	85	78	68 ^*pw*^	63	385	366	375	839	0.118	4.7
*c*-C_3_N_4_^#215^ [30]	71 ^*pw*^	63 *^pw^*	68 *^pw^*	58 *^pw^*	421 *^pw^*	425	397 ***	908 *^pw^*	0.144 *^pw^*	7.1 ^*pw*^
*c*-C_3_N_4_^#220^ [31]	73 *^pw^*	74 *^pw^*	56 *^pw^*	48 *^pw^*	430 *^pw^*	487 ^§§^	393 ^§§^	930 ^§§^	0.18 ^§§^	9.1 ^*pw*^
*tet*-C_11_N_4_^#115^ [*pw*]	76	78	87	82	408	406	461	1003	0.088	5.5
*tet*-BC_5_^#119^ [32]	70 *^pw^*	62 *^pw^*	68 *^pw^*	62 *^pw^*	350 *^pw^*	376	379	851 *^pw^*	0.123 *^pw^*	4.9 *^pw^*
*c*-BC_7_^#215^ [33]	72 *^pw^*	59 *^pw^*	75 *^pw^*	70 ^*pw*^	358 *^pw^*	375	403	890 *^pw^*	0.104 *^pw^*	4.8 *^pw^*
*trig*-BC_11_^#156^ [34]	83 ^*pw*^	81 ^*pw*^	75 ^*pw*^	68	395 ^*pw*^	405	414	926 ^*pw*^	0.119 ^*pw*^	5.3 ^*pw*^
*o*-BC_2_N ^#17^ [35]	73 ^*pw*^	69 ^*pw*^	88 ^*pw*^	76 ^*pw*^	381 ^*pw*^	459	482	1071 ^*pw*^	0.111 ^*pw*^	6.4 ^*pw*^
*trig*-BC_2_N ^#156^ [35]	74 ^*pw*^	58 ^*pw*^	––	––	384 ^*pw*^	420	––	––	––	––
*rh*-BC_2_N ^#160^ [36]	75	74 ^*pw*^	90	84	426 ^*pw*^	412	476	1031	0.083	5.7
*tet*-BCN ^#105^ [37]	65	61	35 ^*pw*^	36	282	280	232	545	0.175	4.2

* Thermodynamic model [27]; ^†^ Lyakhov–Oganov model [28]; ^‡^ Mazhnik–Oganov model [10]; ^§^ Chen–Niu model [8]; ** *E* and ν values calculated using isotropic approximation; ^††^ Ref. [40]; *** Calculated from elastic moduli C_ij_ [30] using Voigt’s approach [26]; ^§§^ Ref. [41]; *^pw^* present work.

**Table 3:** Vickers hardness of hypothetical ternary B–C–N phases calculated using different models.

	*H*_V_ (GPa)		
	Gao’s Model *	Tian’s Model ^†^	Thermodynamic Model ^‡^
*c*-BC_2_N [63]	79.7	–	75 ^*pw*^
*trig*-BC_4_N (*P*3*m*1) [65]	84.3	–	76 ^*pw*^
*o*-BC_4_N (*Imm*2) [66]	78.7	–	73 ^*pw*^
*trig*-BC_4_N [67]	–	87.5	73 ^*pw*^
*tet*-BC_6_N (*P*−42*m*) [68]	79.9	–	75 ^*pw*^
*rh*-BC_6_N (*R*3*m*) [68]	79.1	–	76 ^*pw*^
*m*-BC_6_N (*Pm*) [69]	77.4	–	72 ^*pw*^
*m*-BC_6_N (*Cm*) [69]	80.6	–	73 ^*pw*^
*trig*-BC_10_N (*P*3*m*1) [70]	–	87	75 ^*pw*^
*tet*-BCN (*I*4_1_*md*) [64]	61.8	–	70 ^*pw*^
*trig*-BCN (*P*−3*m*1) [64]	68.5	–	69 ^*pw*^

* Ref. [71]; ^†^ Ref. [9]; ^‡^ Ref. [27]; *^pw^* present work.

## Data Availability

The data presented in this study are available on request.

## Appendix A. Computational Framework

Accurate energy-based studies are primarily achieved within the framework of quantum mechanics. The most successful framework is density functional theory (DFT), whose theoretical basis was presented by Hohenberg and Kohn in 1964 [74]. One year later, Kohn and Sham devised the so-called KS equations to efficiently solve the wave equation of the system [75] using computational codes based on DFT. Among the numerous programs, we used the plane-wave Vienna Ab Initio Simulation Package (VASP) code [76,77] with the projector augmented wave (PAW) method [77,78] for the atomic potentials with all valence states, especially in regard to light elements such as boron, carbon and nitrogen. The exchange-correlation effects inherent to DFT were considered with the generalized gradient approximation (GGA) following Perdew, Burke and Ernzerhof (PBE) [79]. The PBE scheme was affirmed by Kilmes et al. as the best suited for quantum calculations of carbon structures [80]. A conjugate-gradient algorithm [81] was used in this computational scheme to relax the atoms onto the ground state. The tetrahedron method with Blöchl et al. corrections [82] and the Methfessel–Paxton scheme [83] were applied for both geometry relaxation and total energy calculations, respectively. Brillouin-zone integrals were approximated using a special **k**-point sampling scheme by Monkhorst and Pack [84]. The optimization of the structural parameters was performed until the forces on the atoms were less than 0.02 eV/Å, and all stress components below 0.003 eV/Å^3^. The calculations were converged at an energy cut-off of 500 eV for the plane-wave basis set concerning the **k**-point integration in the Brillouin zone, with a starting mesh of 6 × 6 × 6 up to 12 × 12 × 12 for the best convergence and relaxation to zero strains. In the post-treatment process of the ground state electronic structures, the electron localization function and the electronic and phonon band structures were computed, visualized and assessed. Calculations of phonon dispersion curves were also carried out to verify the dynamic stability of the proposed structures. The phonon modes were computed via finite displacements of the atoms off their equilibrium positions to obtain the forces from the summation over different configurations. The phonon dispersion curves along the direction of the Brillouin zone were subsequently obtained using the “phonopy” code based on the Python language [85].

Further investigations of the electronic band structure and electron density of states were carried out with the full-potential DFT-built augmented spherical-wave ASW method [86]. Figure 4c,d show the results of band structure calculations for template diamond-like *hex*-C_12_ and the resulting trigonal BC_11_.
